# Supplementary figures and images for: A Role for the V0 Sector of the V-ATPase in Neuroexocytosis: Exogenous V0d Blocks Complexin and SNARE Interactions with V0c
Source: Cells. 2023 Feb 26;12(5):750. doi: 10.3390/cells12050750 (PMC10001230; doi:10.3390/cells12050750)

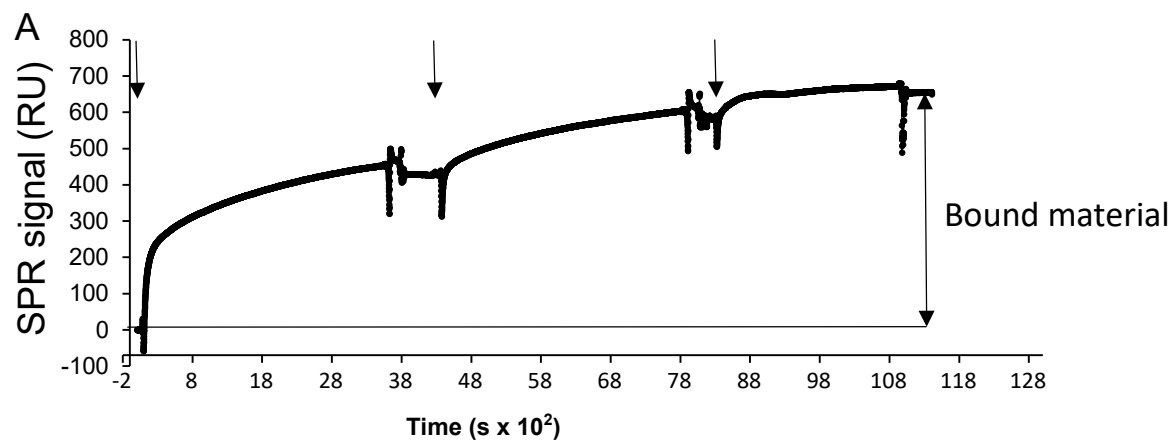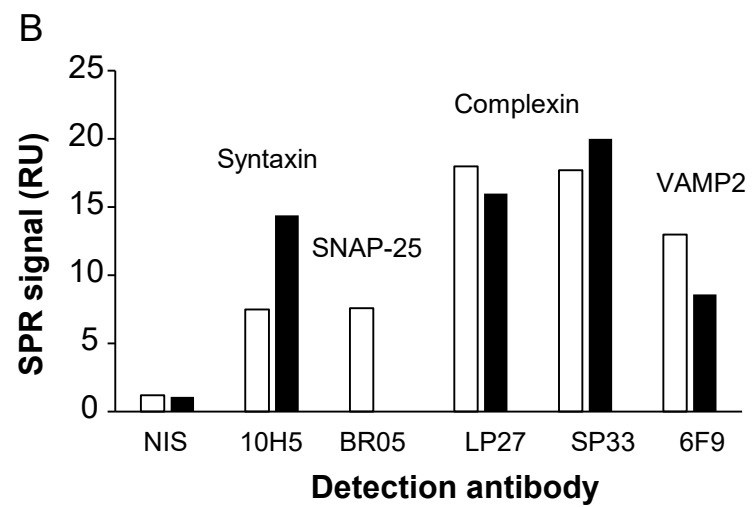

Supplement: Supplementary file 1 [file cells-12-00750-s001.zip › Figure S1.pdf]

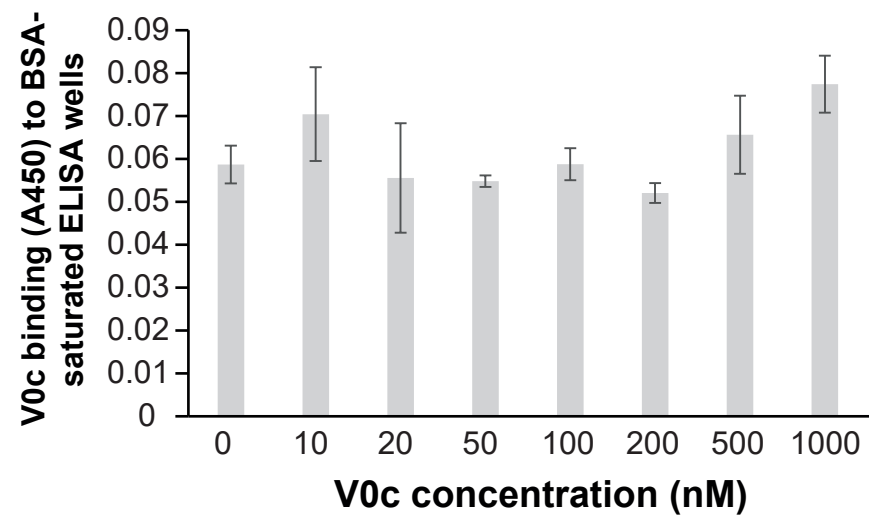

Supplement: Supplementary file 1 [file cells-12-00750-s001.zip › Figure S2.pdf]

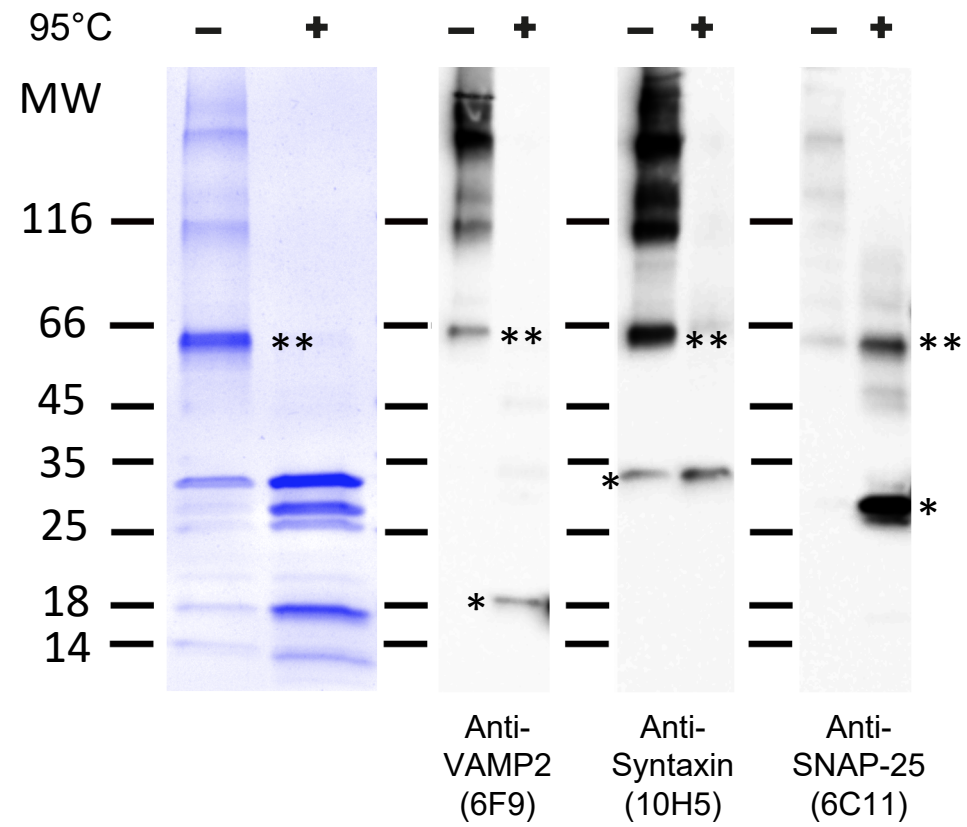

Supplement: Supplementary file 1 [file cells-12-00750-s001.zip › Figure S3.pdf]

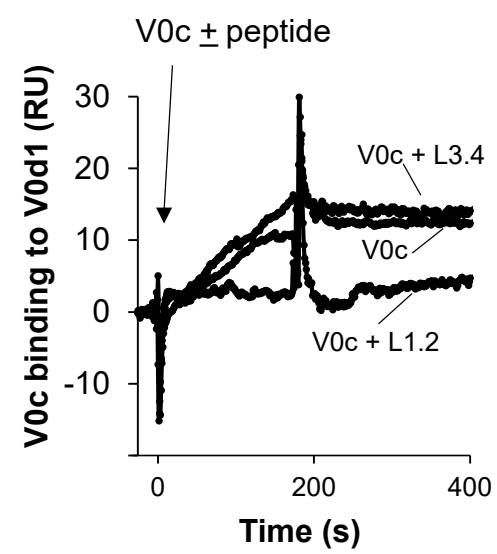

Supplement: Supplementary file 1 [file cells-12-00750-s001.zip › Figure S4.pdf]

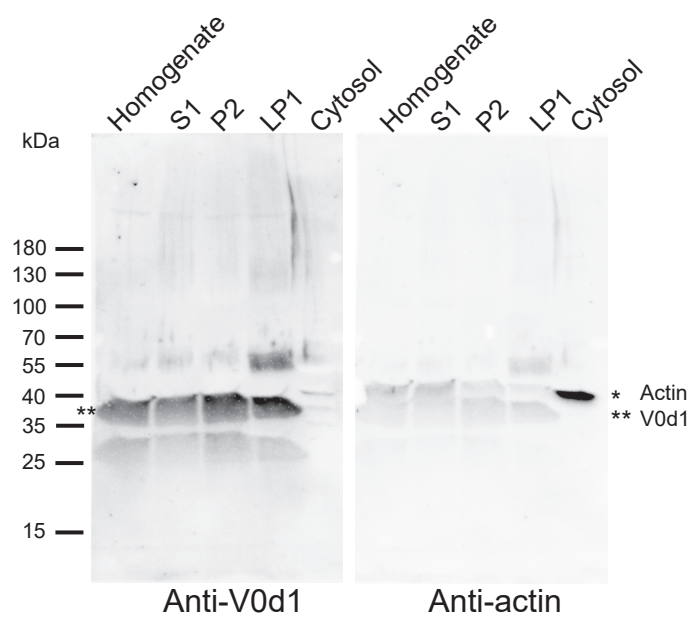

Supplement: Supplementary file 1 [file cells-12-00750-s001.zip › Figure S5.pdf]

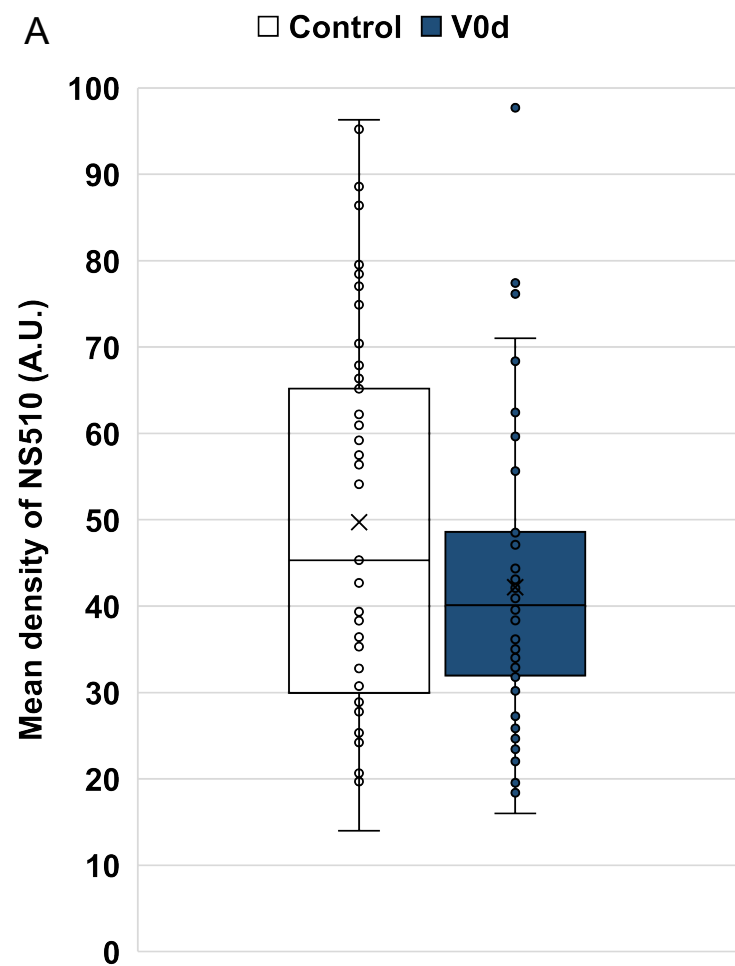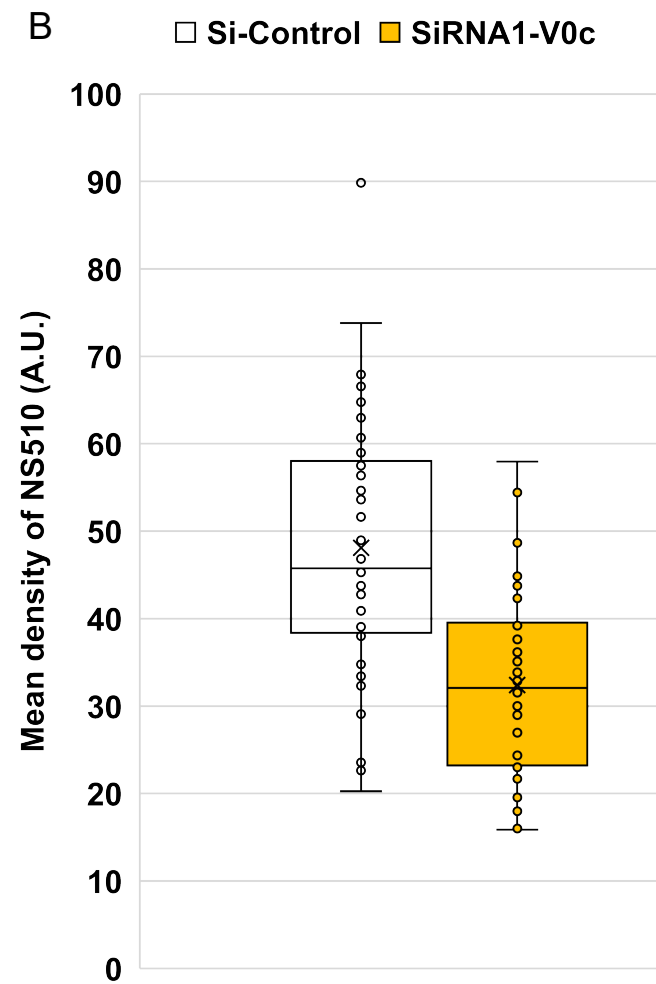

Supplement: Supplementary file 1 [file cells-12-00750-s001.zip › Figure S6.pdf]
